# Synthesis of a New Series of Pyridine and Fused Pyridine Derivatives

**DOI:** 10.3390/molecules170910902

**Published:** 2012-09-11

**Authors:** Siham AbdulRahman Al-Issa

**Affiliations:** Chemistry Department, Faculty of Science, Princess Nora Bint Abdul Rahman University, Riyadh 11435, Saudi Arabia; Email: sehamelissa@yahoo.com

**Keywords:** pyridine derivatives, pyrido-[2,3-d]-pyrimidine, pyrazolo-[3,4-b]-pyridine

## Abstract

The reaction of 4-methyl-2-phenyl-1,2-dihydro-6-oxo-5-pyridine- carbonitrile (**1**) with arylidene malononitrile afforded isoquinoline derivatives **2a**,**b**. 6-Chloro-4-methyl-2-phenyl-5-pyridinecarbonitile (**3**) obtained by chlorination of compound **1** with phosphoryl chloride was converted into 6-amino-4-methyl-2-phenyl-5-pyridinecarbonitrile (**4**) and 6-hydrazido-4-methyl-2-phenyl-5-pyridinecarbonitrile (**5**) in good yield, through reactions with ammonium acetate and hydrazine hydrate, respectively. Treatment of **4** with ethyl acetoacetate, acetic anhydride, formic acid, urea and thiourea gave the corresponding pyrido [2,3-d] pyrimidine derivatives **7**–**10a**,**b**. A new series of 6-substituted-4-methyl-2-phenyl-5-pyridine carbonitriles **11**–**13** has been synthesized via reaction of **4** with phenyl isothiocyanate, benzenesulphonyl chloride and acetic anhydride. Treatment of **4** with malononitrile gave 1,8-naphthyridine derivative **14**. The reactivity of hydrazide **5** towards acetic acid, phenylisothiocyanate and methylacrylate to give pyrazolo-[3,4-b]-pyridine derivatives **15**–**17** was studied. Treatment of **5** with acetic anhydride, phthalic anhydride and carbon disulphide gave pyridine derivatives **18**,**19** and 1,2,4-triazolo-[3,4-a]-pyridine derivative **20**.

## 1. Introduction

A large number of heterocyclic compounds containing pyridine rings are associated with diverse pharmacological properties such as antimicrobial [1,2], anticancer [3] anticonvulsant [4], antiviral [5], anti-HlV [6], antifungal and antimycobacterial activities [7]. Pyrido-[2,3-d]-pyrimidines are heterocyclic ring systems of considerable interest due to several biological activities associated with this scaffold. Some analogues have been found to act as antitumor agents inhibiting dihydrofolate reductases or tyrosine kinases [8,9], while other are known antiviral agents [10]. The pyrazolo-[3,4-b]-pyridine framework is a key structural fragment of many heterocyclic compounds showing a broad spectrum of biological activity [11]. In the last decade, some heterocycles of this class have been found to regulate the cardiovascular system and possess antiviral [5,12,13], antileishmanial [14] and antimicrobial properties [15].

Prompted by recent literature observations, some new pyridine derivatives were synthesized, leading to interesting heterocyclic scaffolds that are particularly useful for the creation of diverse chemical libraries of drug-like molecules for biological screening.

## 2. Results and Discussion

**Scheme 1:**
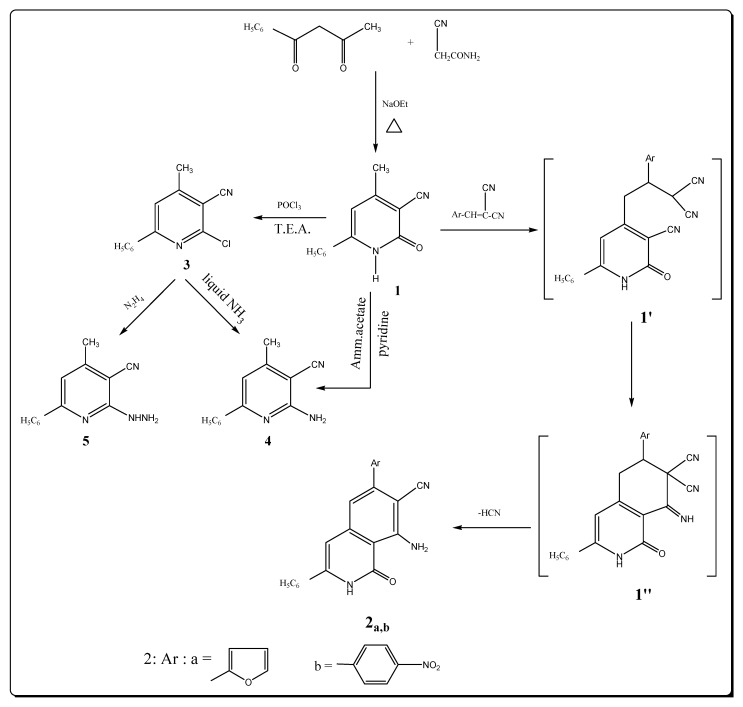
Synthesis of isoqunoline and pyridine derivatives.

The starting material pyridinecarbonitrile **1** was prepared by condensing benzoylacetone with cyanoacetamide in the presence of sodium ethoxide [16]. The reaction of **1** with arylidenmalononitriles afforded the expected isoquinoline derivatives **2a**,**b** (Scheme 1) most probably involving intermediate adduct **1′**, which then cyclized into **1′′**. Final loss of HCN yields aromatic **2a**,**b**. The appearance of bands at 3434, 3329, 3169 attributed to NH_2_ and NH stretching frequency in the IR spectrum of **2a** are good evidence for the structure assigned to this compound; the 2221 cm^–1^ band is due to the presence of the C≡N group, in addition to the C=O group at 1672 cm^–1^. Its ^1^H-NMR spectrum displays specific signals for pyridine protons at position 3 appeared at δ 6.80 ppm, while the singlet due to the NH_2_ protons is observed at δ 4.22 ppm and the absence of a specific CH_3 _signal. The signal of the NH proton is observed at δ 9.80 ppm as a singlet.

Chlorination of cyanopyridone **1** with phosphoryl chloride similar to earlier results [17] gave only a poor yield of impure chlorination products. The addition of triethylamine to the phosphoryl chloride reagent, however, accelerated the reaction rate [18] and afforded 6-chloro-4-methyl-2-phenyl-5-pyridinecarbonitrile (**3**) in good yield, after 4 hours. Compound **3** with a vicinal chloro and cyano groups was envisaged as a potential starting material for the synthesis of fused heterocycle systems. The IR spectrum of compound **3** showed the presence of a nitrile group at 2222 cm^–1^ and revealed the absence of a band characteristic for NH or OH group (see Experimental). The ^1^H-NMR spectrum of **3** displayed a signal at δ 2.44 ppm due to the methyl group, an aromatic multiplet in the δ 7.22–7.48 ppm region, in addition to a signal at 6.80 ppm for the pyridine proton at position 3 and confirmed the absence of a signal characteristic for an OH or NH group. Refluxing **1** in pyridine and ammonium acetate afforded the corresponding 6-amino-4-methyl-2-phenyl-5-pyridinecarbonitrile (**4**) in a good yield. The IR spectrum of compound **4** showed of strong absorption bands of a NH_2_ group at 3350 and 3292 cm^–1^ and a nitrite group at 2220 cm^–1^. The ^1^H-NMR spectrum showed two singlet signals at 2.48 and 4.44 ppm corresponding to the protons of the methyl and amino groups, a singlet at δ 6.80 ppm for the pyridine H-3 proton. The mass spectrum exhibited the molecular ion peak at *m/z* 209 (M^+^, 3.18) corresponding to the formula (C_13_H_11_N_3_) in addition to other peaks at 77 (88%) and 75 (100%). Compound **4** can be obtained in low yield by reaction of compound **3** with cold liquid ammonia.

The pyridine derivative **3** was condensed with hydrazine hydrate to afford the corresponding hydrazide **5**. The IR and ^1^H-NMR of compound **5** revealed that this hydrazide was present in acyclic form. The ^1^H-NMR spectrum showed broad singlets at δ 5.14 and 9.22 ppm corresponding to the hydrazide NH_2_ and NH groups, as well as the characteristic absorption band at 3428 and 3298 cm^–1^ and nitrile group at 2220 cm^−1^ in its IR spectrum. The mass spectrum exhibited the molecular ion peak at *m/z* 244 (M^+^, 2.11) and base peak at 75 (100%).

**Scheme 2:**
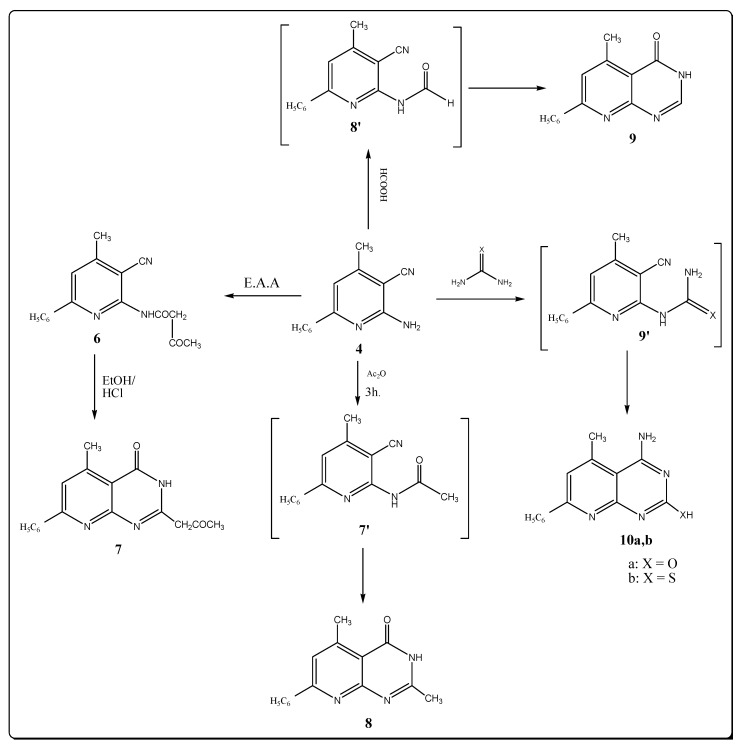
Synthesis of pyridopyrimidine derivatives.

The target pyrido [2,3-d] pyrimidine-4-one derivatives were synthesized by the reactions depicted in Scheme 2. Thus reacting compound **4** with ethyl acetoacetate by fusion for one hour afforded the desired carboxamide **6** in excellent yield. No evidences for the presence of an amino (NH_2_) group was seen in both the IR and ^1^H-NMR spectra. Cyclization of a cyclic amide keto function with the nitrile group at position 3 by refluxing **6** in ethanol with hydrochloric acid produced 5-methyl-7-phenyl-2-propanone-3*H*-pyrido-[2,3-d]-pyrimidine-4-one (**7**). The IR spectra of **7** contains bands at 3299, 1686 and 1660 cm^−1^ corresponding to (NH) group, and the amide and ketone carbonyl groups. The ^1^H-NMR spectrum exhibits a broadened signlet at δ 12.34 belonging to the (NH) group. The ^13^C-NMR spectra of carboxamide **6** and pyrido-[2,3-d]-pyrimidin-4-one **7** agree with their assigned structures.

Thermal cyclocondensation of 2-aminopyridine derivative **4** with acetic anhydride and formic acid afforded pyrido-[2,3-d]-pyrimidine-4-one derivatives **8** and **9**. This reaction was carried out thermally in the absence of solvent. The structure of compounds **8** and **9** were inferred from their IR, ^1^H-NMR spectral data and elemental microanalysis. The spectral data of the products **8** and **9** revealed the disappearance of the NH_2_ group, indicating their involvement in the cyclization process. The characteristic IR stretching band at 1680 and 1688 cm^–1^ shows the occurrence of (C=O) groups due to the pyridopyrimidinone systems. Formation of the compounds **8** and **9** is thought to occur through the expected intermediates **7′** and **8′** which were not isolated (Scheme 2). The ^1^H-NMR spectrum of compound **8** revealed two characteristic singlet signals at δ 2.50 and 11.82 ppm due to methyl protons at C-2 of a pyrimidine moiety and a NH proton, respectively, but the ^1^H-NMR of compound **9** revealed the presence of a singlet signal at 8.28 ppm due to the proton at position 2 in the pyrimidine ring. When compound **4 **was treated with urea or thiourea by fusion, it furnished products identified as 4-amino-5-methyl-7-phenyl-(1*H*)-pyrido-[2,3-d]-pyrimidine-2-one (or thione) **10a**,**b**. The IR spectra of **10a**,**b** showed the disappearance of the C≡N group and showed absorptions bands at 3320, 3250, 3330, 3280 for NH_2_ groups. The ^1^H-NMR spectra exhibited broad signals at δ 8.91 and 10.68 ppm due to NH protons.

**Scheme 3:**
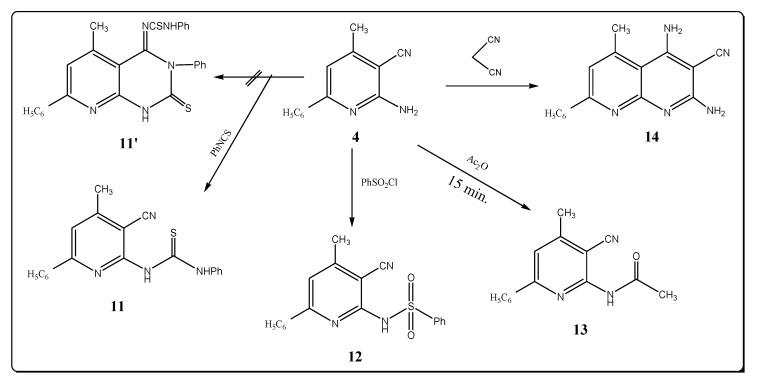
Synthesis of pyridine derivatives.

Moreover, compound **4**, upon treatment with phenyl isothiocyanate afforded the corresponding thiourea derivative **11**. The spectral data are in agreement with the assigned structure (Scheme 3). Thus the IR spectrum of compound **11** shows the presence of nitrile and NH stretching bands near 2222 and 3212 cm^–1^, respectively, and revealed the lack of a characteristic amino group band (see Experimental). The ^1^H-NMR spectrum of compound **11** showed two singlet signals at δ 11.08 and 13.26 ppm due to the protons at two (NH) groups, in addition to a MS peak at *m/z* = 344 (M^+^, 2.18). The possibility of formation of the product **11′** is excluded on the basis of the spectral data.

The reaction of compound **4** with benzenesulfonyl chloride led to the formation of 6-benzene sulfonylamino-4-methyl-2-phenyl-5-pyridinecarbonitile (**12**). IR spectrum of **12** displayed absorption bands at 3250, 2215, 1360 and 1150 cm^–1^ which are characteristic for NH, C≡N and SO_2_ asym, SO_2_ sym groups. The ^1^H-NMR spectrum of **12** showed a signal at δ 8.50 ppm for the proton of a (SO_2_NH) group. However, upon heating compound **4** in refluxing acetic anhydride for 15 min. it afforded 6-acetylamino-4-methyl-2-phenyl-5-pyridinecarbonitrile (**13**). IR spectrum of **13** showed absorption bands at 3280, 2220 and 1720 cm^–1^ which are characteristic of NH, C≡N and C=O groups. The ^1^H-NMR spectrum of **13** showed two singlet signals at δ 2.50 and 3.33 ppm corresponding to the protons of two methyl groups, and a singlet at δ 10.22 ppm for a (NH) group. Treatment of compound **4** with malonitrile in refluxing ethanol in the presence of triethylamine gave 2,4-diamino-3-cyano-5-methyl-7-phenyl-1,8-naphthyridine (**14**), whose structure is supported by elemental analysis, its IR spectrum, which showed bands at 3450, 3400, 3340, 3250 cm^–1^ (2NH_2_) and 2230 cm^–1^ (C≡N) and the ^1^H-NMR spectrum that showed two singlet at δ 4.42, 5.33 ppm for two amino groups.

**Scheme 4:**
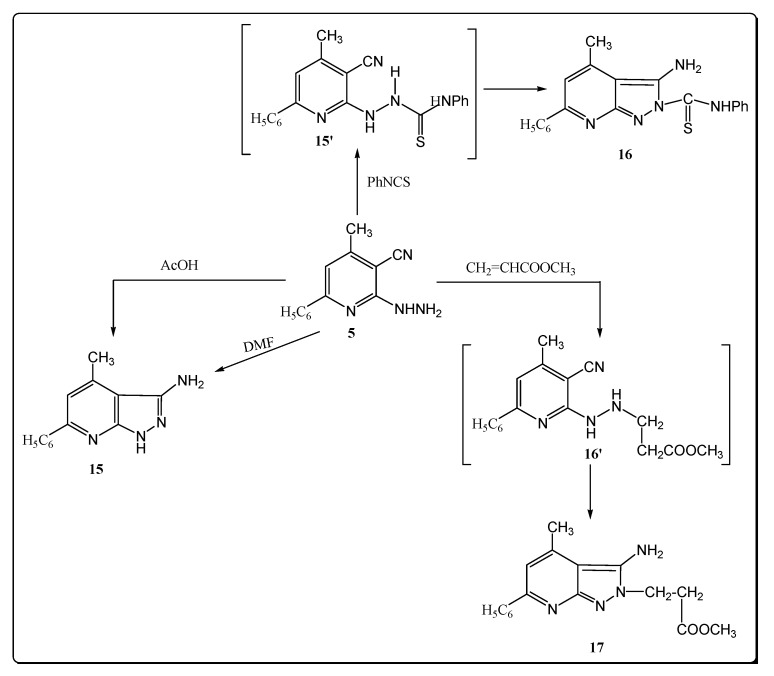
Synthesis of pyrazolo pyridine derivatives.

Hydrazide derivative 5 proved to be versatile compounds by virtue of its vicinal cyano functions as confirmed by its reactivity in several cyclization reaction performed with the aim of obtaining new heterocycles with a conserved pyrazolo-[3,4-b]-pyridine motif (Scheme 4).

Thus, reaction of compound 5 with acetic acid afforded the corresponding 3-amino-4-methyl-6-phenyl-1*H*-pyrazolo-[3,4-b]-pyridine (**15**), through intramolecular cyclization via addition of the NH_2_ functional group at the C≡N group. The proposed structure for **15** was supported by its independent synthesis from **5** by refluxing with DMF (melting point and mixed melting point was not depressed) (Scheme 4). The IR spectrum of compound **15** showed three absorption bands at 3320, 3300, 3221 cm^−1^ assigned to the stretching vibrations of NH_2_ and NH groups, respectively. The ^1^H-NMR spectrum revealed two singlet signals at δ 4.50 and 12.11 ppm corresponding to NH_2_ and NH groups. In addition, the mass spectrum of **15** showed a peak at *m/z* 244 (M^+^, 6.88) corresponding to its molecular ion.

Interestingly, compound **5 **seemed to be a useful candidate for further chemical transformations. Thus, the reaction of **5** with phenyl isothiocyanate in absolute ethanol at refluxing temperature afforded the corresponding thiosemicarbazide derivatives **15′**, which underwent a further an intramolecular cyclization to afford 3-amino-4-methyl-6-phenyl-1*H*-pyrazolo-[3,4-b]-pyridine-2-yl-phenyl thioamide (**16**), identified by the absence of the cyano group signal in its IR and the presence of a signal of the amino group at δ 4.80 ppm and the broad band of the (NHPh) proton at 11.80 ppm in its ^1^H-NMR spectrum, as well as the characteristic absorption band at 3261 cm^–1^ for NH in its IR spectrum. Its mass spectrum showed the molecular ion peak at *m/z* 359 (8.11) and the most intense peak at *m/z* 135, corresponding to [M-PhNCS]^+^.

Treatment of compound **5** with methyl methacrylate in acetic acid at reflux afforded 3-amino-4-methyl-6-phenyl-1*H*-pyrazolo-[3,4-b]-pyridine-2-yl-methyl propionate (**17**). The IR spectrum is characterized by the presence of strong NH_2_ group absorption bands at 3384 and 3280 cm^–1^ and a carbonyl group at 1710 cm^–1^. The ^1^H-NMR spectrum showed a singlet at 2.48 (CH_3_-pyridine), a singlet at 3.38 (OCH_3_), two triplet signals at δ 4.50, 4.80 (2CH_2_ groups). The mass spectrum exhibited the molecular ion peak at *m/z* 310 (M^+^, 0.80) in addition to another peaks at 75 (100).

**Scheme 5:**
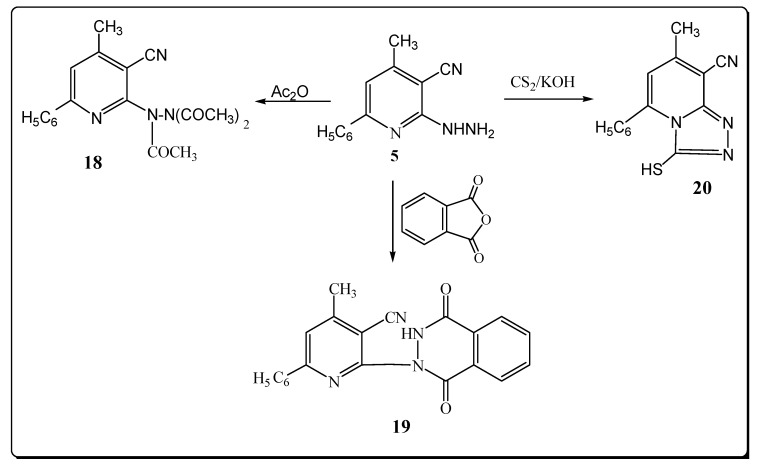
Synthesis of pyridine derivatives.

However, the reaction product upon heating compound **5** in refluxing acetic anhydride was identified as the triacetyl derivative **18** (Scheme 5). The structure of **18** was elucidated from elemental analysis and spectral data. Its IR spectrum showed the absence of a NH_2_ absorption band and the presence of a band at 1681 cm^–1^ (C=O). Its ^1^H-NMR spectrum exhibited signals at 2.50, 2.80, 3.10 for (3COCH_3_).

The mass spectrum exhibited a molecular ion peak at *m/z* 350 (M^+^, 0.82) and a base peak at *m/z* 77 (100). 1-[3-Cyano-4-methyl-6-phenyl]-pyridine-2-yl-(2*H*)-phthalazine-3,8-dione (**19**) was obtained via reaction of compound **5** with phthalic anhydride (Scheme 5). The structure of **19** was elucidated from elemental analysis and spectral data. Its IR spectrum showed the absence of a NH_2_ absorption band and the presence of bands at 3280, 2222 and two bands at 1739, 1714 cm^–1^ due to NH, C≡N and2 C=O functions.

The^ 1^H-NMR revealed a signal for NH at δ 12.21 ppm. The mass spectrum exhibited a molecular ion peak at *m/z* 354 (M^+^, 1.08). When **5** was allowed to react with CS_2_, 8-cyano-7-methyl-5-phenyl-2,3-dihydro-2-thioxo-1,2,4-triazolo-[4,3-a]-pyridine (**20**) was obtained in a good yield (Scheme 5). The structure of **20** was elucidated from elemental analysis and spectral data. Its IR spectrum showed the absence of a NH_2_ absorption band and the presence of bands at 3220 and 2220 cm^–1^ for NH and C≡N groups. The ^1^H-NMR spectrum of compound **20** exhibited a signal at δ 11.33 ppm (NH). The mass spectrum of 20 showed a molecular ion peak at *m/z* 266 (M^+^, 3.11%) with a base peak at *m/z* 75 (100).

## 3. Experimental

### 3.1. General

All melting points were recorded on a Gallenkamp melting apparatus and are uncorrected. The IR spectra were recorded on a Pye-Unicam Sp-3-100 spectrophotometer using the KBr wafer technique. ^1^H-NMR and ^13^C-NMR spectra were recorded on a Bruker 400 MHz instrument with DMSO-d_6_ as solvent and tetramethylsilane as an internal standard; chemical shifts are as δ units (ppm). The mass spectra were recorded on a MS-S988 instrument operating at 70 eV. Elemental analysis was determined using a Perkin-Elmer 240C microanalyser.

### 3.2. Syntheses

*8-Amino-7-cyano-1-oxo-6-aryl-3-phenyl-1,2-dihydroisoquinolines* (**2a**,**b**). A solution of compound **1** (10 mmol) in pyridine (30 mL) was treated with an arylidene malononitrile (10 mmol). The reaction mixture was refluxed for 6 h, left to cool to room temperature, poured into ice-cold water, and neutralized with HCl (10%). The solid product was filtered off and recrystallized from ethanol. **2a**: Yield 60%; m.p. > 360 °C; IR cm^–1^: 3434, 3329 (NH_2_) 3169 (NH), 3050 (CH–Ar), 2221 (C≡N), 1672 (C=O); ^1^H-NMR: δ 4.22 (s, 2H, NH_2_), 6.80 (s, 1H, pyridine-H) 7.22–7.72 (m, 9H, Ar–H), 9.80 (s, 1H, NH); ^13^C-NMR: δ 115.00 (C≡N), 188.00 (C=O), 111.00–142.90 (Ar–C); MS *m/z* (%): 327 (M^+^, 100). Anal. For: C_20_H_13_N_3_O_2_: Calcd. C, 73.39; H, 3.97; N, 12.84 Found: C, 73.40, H, 3.88; N, 13.00. **2b**: Yield 62%; m.p. 280 °C; IR cm^–1^: 3430, 3333 (NH_2_) 3199 (NH), 3050 (CH–Ar), 2215(CN), 1666 (C=O); ^1^H-NMR: 4.82 (s, 2H, NH_2_), 6.64 (s, 1H, pyridine-H-3) 7.88 (d, 2H, Ar–H, *J* = 7.00 Hz) 9.12 (d, 2H, Ar–H, *J* = 8.22 Hz), 7.64 (m, 6H, Ar–H), 9.30 (s, 1H, NH); ^13^C-NMR: δ 118.00 (C≡N), 188.00 (C=O), 101.00-139.63 (Ar-C); MS *m/z* (%): 382 (M^+^, 8.11), (77, 100). Anal. For: C_22_H_14_N_4_O_3_: Calcd. C, 69.10; H, 3.66; N, 14.65; Found: C, 69.30, H, 3.80; N, 14.80.

*6-Chloro-4-methyl-2-phenyl-5-pyridinecarbonitrile* (**3**). A mixture of **1** (116 mmol) and phosphorus oxychloride (55.9 mL, 600 mmol) in the presence of triethylamine (3 mL) was stirred at 105 °C for 3 h. The mixture was poured onto crushed ice (800 g) and neutralized with 10% sodium hydroxide. The extract was washed with water 5% aqueous sodium bicarbonate and again with water and recrystallized from ethanol. **3**: Yield 72%, m.p. 180–182 °C, IR cm^–1^: 3033 (CH–Ar), 2999 (CH–aliph.), 2222 (C≡N), 1644 (C=N); ^1^H-NMR: δ 2.44 (s, 3H, CH_3_), 6.80 (s, 1H, pyridine-H-3), 7.22–7.48 (m, 5H, Ar–H); ^13^C-NMR: δ 116.22 (C≡N), 128.34–148.00 (Ar–C); MS *m/z* (%): 228.5 (3.28), 230.5 (1.11), 57 (100). Anal. For: C_13_H_9_ClN_2_: Calcd. C, 68.27, H, 3.93, N, 12.25, Cl, 15.53. Found. C, 68.30; H, 2.00; N, 12.50; Cl, 15.98.

*6-Amino-4-methyl-2-phenyl-5-pyridinecarbonitrile* (**4**). Method A: A mixture of compound **1** (0.005 mL) and ammonium acetate (0.005 mol) in pyridine (30 mL) was refluxed for 8 h. The reaction mixture was cooled at room temperature, poured into ice water, and neutralized with dilute HCl. The isolated solid was filtered off and recrystallized from ethanol to afford **4**: Yield 75%; m.p. 250–252 °C; IR cm^–1^: 3350, 3292 (NH_2_), 3100 (CH–Ar), 2900 (CH–aliph.), 2220 (C≡N), 1644 (C=N); ^1^H-NMR: δ 2.48 (s, 3H, CH_3_), 4.44 (s, 2H, NH_2_), 6.80 (s, 1H, pyridine-H-3), 7.22–7.68 (m, 5H, Ar–H); ^13^C-NMR: δ 115.72 (C≡N), 122.42–152.23 (Ar–C); MS *m/z* (%): 209 (M^+^, 3.18), 75 (100). Anal. For: C_13_H_11_N_3_: Calcd. C, 74.64; H, 5.26; N, 20.09; Found: C, 74.71, H, 5.22, N, 20.38. Method B: To a solution of compound **3** (9.58 mmL) in DMF (25 mL) was added NH_3 _ (50 mL, 25% in water) and the resulting mixture was stirred at room temperature for 12 h. The mixture was extracted with ethylacetate (3 × 50 mL) and the combined organic layer was dried over Na_2_SO_4_, filtered and concentrated under vacuum and the resulting solid was recrystallized from ethanol to afford **4** in a low yield.

*6-Hydrazide-4-methyl-2-phenyl-5-pyridinecarbonitrile* (**5**). A mixture of compound **3** (0.01 mmol) and hydrazine hydrate (98%, 0.01 mmol) in ethanol (20 mL) was heated under reflux for 6 h. The excess of solvent was removed under reduced pressure, and the resulting precipitate was filtered off, washed with ethanol and recrystallized from methanol to give **5**, yield 62%; m.p. 100–102 °C; IR cm^–1^; 3428–3298 (NH, NH_2_), 3046 (CH–Ar), 2921 (CH–aliph.), 2220 (C≡N), 1632 (C=N); ^1^H-NMR: δ 2.43 (s, 3H, CH_3_), 5.14 (br. S, 2H, NHNH_2_), 6.71 (s, 1H, pyridine-H-3), 7.33–7.80 (m, 5H, Ar–H), 9.12 (s, 1H, NH), ^13^C-NMR: δ 118.64 (C≡N), 126.88–155.43 (Ar–C); MS *m/z* (%): 244 (M^+^, 2.11), 75 (100). Anal. For: C_13_H_12_N_4_: Calcd. C, 69.64; H, 5.35; N, 25.00; Found: 69.70, H, 5.50; N, 24.80.

*2-(3-Cyano-4-methyl-6-phenyl-pyridine)-2,4-dioxo butanamide* (**6**). A mixture of compound **4** (5.62 mmol) and (8.00 mL) of ethyl acetoacetate was heated for 6 h at 140–150 °C. Excess ethyl aceto- acetate was distilled off under reduced pressure, and the residue was purified by recrystallization from ethanol. **6**: Yield 75%; m.p. 110–112 °C; IR cm^–1^: 3210 (NH), 3032 (CH–Ar), 2990–2828 (CH–aliph.), 2215 (C≡N) 1710 (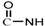), 1680 (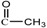); ^1^H-NMR: δ 2.48 (s, 3H, CH_3_), 3.80 (s, 3H, CH_3_), 4.24 (s, 2H, CH_2_), 7.12–7.48 (m, 6H, Ar–H), 11.55 (s, 1H, NH); ^13^C-NMR: δ 14.11 (CH_3_), 64.19 (CH_2_), 115.00 (C≡N), 112.88–156.09 (Ar–C), 168.50 (CO–CH_3_), 182.41 (CO–NH); MS *m/z* (%): 293 (9.80), 75 (100). Anal. For: C_17_H_15_N_3_O_2_: C, 69.62; H, 5.11; N, 14.33; Found: C, 69.60, H, 5.22, N, 14.50.

*5-Methyl-7-phenyl-2-propanone-3H-pyrido [2,3-d] pyrimidine-4-one* (**7**). Compound **6** (0.01 mol) was treated with concentrated hydrochloric acid (3 drops) in ethanol (30 mL) and refluxed for 6 h. The reaction mixture was left to cool, and then poured into ice-cold water. The precipitate was collected and recrystallized from ethanol. **7**: Yield 55%; m.p. 222–224 °C, IR cm^–1^: 3299 (NH), 3112 (CH–Ar), 2989–2885 (CH–aliph.), 1686 (C=O) 1660 (C=O); ^1^H-NMR: δ 2.48 (s, 3H, CH_3_), 3.11 (s, 3H, COCH_3_), 4.17 (s, 2H, CH_2_), 7.22–7.78 (m, 6H, Ar–H), 12.34 (br s, 1H, NH); ^13^C–NMR: δ 14.36 (CH_3_), 16.50 (COCH_3_), 64.04 (CH_2_), 112.69–156.64 (Ar–C), 169.80 (C=O), 189.00 (C=O). MS *m/z* (%): 393 (M^+^, 4.12), (74, 100). Anal. For: C_17_H_15_N_3_O_2_: Calcd. C, 69.62; H, 5.11; N, 14.33; Found: C, 69.50, H, 5.10, N, 14.30.

*2,5-Dimethyl-7-phenyl-3H-pyrido[2,3-d] pyrimidine-4-one* (**8**). Compound **4** (0.002 mol) in acetic anhydride (10 mL) was heated under reflux for 3 h. After cooling, the solvent was concentrated under reduced pressure, then the reaction mixture was poured into ice-water (40 mL) to give a solid precipitate which was filtered off and recrystallized from petroleum ether 60/80 to furnish **8**, yield 55%; m.p. 300–302 °C; IR cm^–1^: 3300 (NH), 3033 (CH–Ar), 2928 (CH–aliph.) 1680 (C=O), 1644 (C=N); ^1^H-NMR: δ 2.50, 2.44 (2s, 6H, 2CH_3_), 7.38–7.80 (m, 6H, Ar–H), 11.82 (s, 1H, NH); ^13^C-NMR: δ 13.02 (CH_3_), 14.22 (CH_3_), 112.70–148.12 (Ar–C), 170.80 (C=O); MS *m/z* (%): 251 (2.11), 77 (100). Anal. For: C_15_H_13_N_3_O: Calcd. C, 71.71; H, 5.17, N, 16.73; Found: C, 71.50, H, 5.20, N, 16.80.

*5-Methyl-7-phenyl-3H-pyrido [2,3-d] pyrimidin-4-one* (**9**). Compound **4** (0.002 mol) in formic acid (10 mL) was heated under reflux for 6 h. After cooling the reaction mixture was poured into ice-water (40 mL) to give a solid precipitate which was filtered off and recrystallized from methanol to furnish **9**, yield 45%; m.p. 180–182 °C; IR cm^–1^: 3330 (NH), 3100 (CH–Ar), 2900 (CH–aliph.) 1688 (C=O), 1640 (C=N); ^1^H-NMR: δ 2.55 (s, 3H, CH_3_), 6.80 (s, 1H, pyridine-H-6), 7.28-7.84 (m, 5H, Ar-H), 8.28 (s, 1H, pyrimidine-H-2). ^13^C-NMR: δ 14.62 (CH_3_), 110.18–155.80 (C–Ar), 188.01 (C=O); MS *m/z* (%): 237 (5.18) 75 (100). Anal. For: C_14_H_11_N_3_O: Calcd. C, 70.88; H, 4.64; N, 17.72. Found: C, 70.80; H, 4.70, N, 17.80.

*4-Amino-5-methyl-7-phenyl-1H-pyrido [2,3-d] pyrimidine-2-one(or thione)* (**10a**,**b**). A mixture of **4** (0.01 mol) and urea (or thiourea) (0.01 mol) was fused in an oil bath at 180 C, for 1h. After cooling and dilution with ethanol (30 mL) the solid product formed was filtered off and recrystallized from ethanol. **10a**: Yield 70%; m.p. 320–322 °C; IR cm^–1^: 3483, 3320, 3250 (OH) (NH_2_, NH), 3033 (CH–Ar), 2880 (CH–aliph.), 1683 (C=O), 1638 (C=N); ^1^H-NMR: δ 2.44 (s, 3H, CH_3_), 4.48 (s, 2H, NH_2_), 6.88–7.44 (m, 6H, Ar–H), 8.91 (s, 1H, NH); ^13^C-NMR: δ 11.12 (CH_3_), 202.32 (C=O), 112.43–148.91 (Ar–C); MS m/z (%): 252 (3.21) 75 (100). Anal. For: C_14_H_12_N_4_O: Calcd. C, 66.66; H, 4.76; N, 22.22; Found: C, 66.50; H, 4.80, N, 22.50. **10b**: Yield 65%; 300–302 °C; IR cm^–1^: 3330, 3280, 3221 (NH_2_, NH), 3052 (CH–Ar), 2988 (CH–aliph.), 1222 (C=S); ^1^H-NMR: δ 2.50 (s, 3H, CH_3_), 4.22 (s, 2H, NH_2_) 6.88–7.22 (m, 6H, Ar–H), 10.68 (s, 1H, NH); ^13^C-NMR: δ 13.43 (CH_3_), 212.98 (C=S), 112.65–144.34 (Ar–C); MS *m/z* (%): 268 (1.11), 77 (100). Anal. For: C_14_H_12_N_4_S: Calcd., C, 62.68; H, 4.47; N, 20.89, S, 11.94. Found: C, 62.80; H, 4.50, N, 2.80, S, 12.12.

*N-(Phenyl)-N′-[2(3-cyano-4-methylphenylpyridinyl] thiourea* (**11**). A mixture of compound **4** (0.01 mol), finely divided sodium metal (0.01 mol) and phenyl isothiocyanate (0.01 mol) were refluxed for 6 h. in dry dioxane (50 mL). After cooling, the solvent was concentrated under pressure, then the reaction mixture was poured into ice-water (40 mL) to give a solid precipitate which was filtered off and recrystallized from petroleum ether 60/80 to furnish **11**, yield 60%; m.p. 220–222 °C. IR cm^–1^: 3212 (NH), 2222 (CN); ^1^H-NMR: δ 2.55 (s, 3H, CH_3_), 11.08 (br. s, 1H, NH), 13.26 (br. s, 1H, NH), 7.58–7.69 (m, 5H, Ar–H) ^13^C-NMR: δ 115 (C≡N), 179.30 (C=S), 14.88 (CH_3_), 112.00–155.40 (C–Ar); MS *m/z* (%): 344 (M^+^, 2.18). Anal. For: C_20_H_16_N_4_S: Calcd. C, 69.76; H, 4.65; 16.27; S, 9.30; Found: C, 69.50; H, 4.70; N, 16.30, S, 9.50.

*6-Benzenesulfonylamino-4-methyl-2-phenyl-5-pyridinecarbonitrile* (**12**). Compound **4** was taken in a mixture of pyridine (4 mL) and acetic anhydride (20 mL). To the solution formed, *p*-benzenesulphonyl chloride was added (0.01 mol) and the reaction mixture was reflux for 6 h., filtered and poured onto acidic crushed ice. The solid product **1**2 obtained was recrystallized from ethanol. **12**: Yield 55%; m.p. 200–202 °C; IR cm^–1^: 3250 (NH), 3100 (CH–Ar), 2215 (C≡N), 2995 (CH–aliph.), 1632 (C=N), 1360 (S=O, asym), 1150 (S=O, sym); ^1^H-NMR: δ 2.55 (s, 3H, CH_3_); 6.88–7.67 (m, 11H, Ar–H), 8.50 (s, 1H, SO_2_, NH); ^13^C-NMR: δ 14.17 (CH_3_), 117 (C≡N), 110.00–155.50 (Ar–C). Anal. For: C_19_H_15_N_3_O_2_S: calcd. C, 65.32; H, 4.29; N, 12.03; S, 9.16; Found: C, 65.00, H, 4.80; N, 12.00, 55, 9.40.

*6-Acetyl amino-4-methyl-2-phenyl-5-pyridinecarbonitrile* (**13**). A suspension of **4** (0.002 mol) in acetic anhydride (10 mL) was heated under reflux for 15 min. After cooling, the solvent was concentrated under reduced pressure, then the reaction mixture was poured into ice-water (40 mL) to give a solid precipitate which was filtered off and recrystallized from ethanol. **13**: Yield 55%; m.p. 180–182 °C; IR cm^–1^: 4330 (OH), 3280 (NH), 3100 (CH–Ar), 2910 (CH–aliph.), 2220 (C≡N), 1720 (C=O); ^1^H-NMR: δ 2.50 (s, 3H, CH_3_), 3.33 (s, 3H, COCH_3_), 6.82–7.72 (m, 6H, Ar–H), 10.22 (s, 1H, NH); ^13^C-NMR: δ 14.13 (CH_3_), 17.22 (CH_3_), 117 (C≡N), 122.00–156.11 (Ar–C), 168.33 (C=O). Anal. For: C_15_H_13_N_3_O. Calcd.: C, 71.71; H, 5.17; N, 16.73; Found: C, 71: 80; H, 5.00, N, 16.90.

*2,4-Diamino-3-cyano-5-methyl-7-phenyl-1,8-naphthyridine* (**14**). A suspension of **4** (0.01 mol) in ethanol (20 mL) containing a catalytic amount of triethylamine (3 mL) was treated with malononitrile (0.01 mol). The reaction mixture was refluxed for 10 h. The separated solid was filtered off and recrystallized from ethanol. **14**: Yield 72%; m.p. 300–302 °C; IR cm^–1^: 3450, 3400, 3340, 3250 (2NH_2_), 3100 (CH–Ar), 2990 (CH–aliph.), 2230 (C≡N); ^1^H-NMR: δ 2.48 (s, 3H, CH_3_), 4.42, 5.33 (2s, 4H, 2NH_2_) 6.84–7.88 (m, 6H, Ar–H); ^13^C-NMR: δ 13.42 (CH_3_), 118.95 (C≡N), 127.87–149.97 (Ar–C); MS: *m/z* (%): 275 (2.08), 77 (100). Anal. For: C_16_H_13_N_5_: C, 69.81; H, 4.72; N, 25.45; Found: C, 69.50; H, 4.80, N, 25.50.

*3-Amino-4-methyl-6-phenyl-1H-pyrazolo-[3,4-b]-pyridine* (**15**). Method A: A mixture of compound **5** (0.001 mol) in ethanol and few drops of glacial acetic acid was refluxed for 8 h. The reaction mixture was poured into ice-cold water and the solid product was filtered off, washed with petroleum ether and recrystallized from ethanol. Yield 62%; Method B: Compound **5** (0.005 mol) in DMF 20 mL was refluxed for 12 h. and then allowed to cool. The solid product that precipitated on cooling was filtered off, dried and recrystallized from DMF. **15**: Yield 50%; m.p 200–202 °C; IR cm^–1^: 3320, 3300, 3221 (NH_2_, NH), 3090 (CH–Ar), 2980 (CH–aliph.), 1644 (C=N), ^1^H-NMR: δ 2.55 (s, 3H, CH_3_) 4.50 (s, 2H, NH_2_), 7.22–7.76 (m, 6, Ar–H), 12.11 (s, 1H, NH); ^13^C-NMR: δ 11.18(CH_3_), 124.62–149.06 (Ar–C); MS *m/z* (%): 224 (6.88), 75 (100). Anal. For: C_13_H_12_N_4_. Calcd. C, 69.64; H, 5.35; N, 25.00; Found: C, 69.70, H, 5.50, N, 25.32.

*3-Amino-4-methyl-6-phenyl-1H-pyrazolo-[3,4-b]-pyridine-2-yl-phenylthioamide* (**16**). A suspension of 5 (0.001 mol) and phenyl isothiocyanate (0.001 mol) in pyridine (10 mL) was heated under reflux for 6 h. After cooling the reaction mixture was poured into ice/water (30 mL) and neutralized with dilute 10% HCl to give a solid precipitate that was recrystallized from ethanol-DMF (3:1). **16**: Yield 70%; m.p. > 360 °C; IR cm^–1^: 3330, 3280, 3261 (NH_2_, NH), 3055 (CH–Ar), 2990 (CH–aliph.), 1333 (C=S); ^1^H-NMR: δ 2.50 (s, 3H, CH_3_), 4.80 (s, 2H, NH_2_), 6.88–7.76 (m, 11H, Ar–H), 11.80 (br s, 1H, NH); ^13^C-NMR: δ 13.44 (CH_3_), 208.65 (C=S), 114.89–145.67 (Ar–C); MS *m/z* (%): 359 (8.11), 75 (100). Anal. For: C_20_H_17_N_5_S: Calcd. C, 66.85; H, 4.73; N, 19.49; S, 8.91. Found: C, 69.50; H, 5.00, N, 19.11.; S, 9.00.

*3-Amino-4-methyl-6-phenyl-1H-pyrazolo-[3,4-b]-pyridine-2-yl-methyl propionate* (**17**). A mixture of compound **5** (0.01 mol) and methyl methacrylate (0.01 mol) in acetic acid (20 mL) containing 3 drops of concentrated hydrochloric acid was refluxed for 5 h. The reaction mixture was concentrated and allowed to cool and poured onto H_2_O (100 mL). The solid obtained was recrystallized from ethanol. **17**: Yield 45%; m.p. 140–142 °C; IR cm^–1^: 3384, 3280 (NH_2_), 3099 (CH–Ar), 2888–2990 (CH–aliph.), 1710 (C=O); ^1^H-NMR: δ 2.48 (s, 3H, CH_3_), 3.38 (s, 3H, OCH_3_) 4.50 (t, 2H, CH_2_, *J* = 10.11 Hz), 4.80 (t, 2H, CH_2_, *J* = 8.22 Hz) 5.80 (s, 2H, NH_2_), 6.82–7.78 (m, 6H, Ar–H); ^13^C-NMR: δ 14.13 (CH_3_), 17.80 (OCH_3_) 40.81 (CH_2_), 60.38 (CH_2_), 112.8–153.11 (Ar–C); MS *m/z* (%): 310 (M^+^, 0.80) 75 (100). Anal. For: C_17_H_18_N_4_O_2_: Calcd. C, 65.80; H, 5.80; N, 18.06, Found: C, 65.38; H, 9.13, N, 18.00.

*4-Methyl-2-phenyl-6-triacetylhydrazide-5-pyridinecarbonitrile* (**18**). Compound **5** (0.002 mol) in acetic anhydride (10 mL) was heated under reflux for 1h. After cooling, the solvent was concentrated under reduced pressure, then the reaction mixture was poured into ice-water (40 mL) to give a solid precipitate which was filtered off an recrystallized from methanol. **18**: Yield 60%; m.p. 180–182 °C; IR cm^–1^: 3100 (CH–Ar), 2910 (CH–aliph.), 2215 (C≡N), 1681 (C=O); ^1^H-NMR: δ 2.48 (s, 1H, CH_3_), 2.50, 2.80, 3.10 (3s, 9H, COCH_3_) 7.52–7.78 (m, 6H, Ar–H), ^13^C-NMR: δ 13.22 (CH_3_) 17.11 (COCH_3_), 19.28 (COCH_3_), 21.00 (COCH_3_) 115 (C≡N), 186.00 (C=O), 180.11 (C=O), 110.66–153.18 (Ar–C); MS *m/z* (%): 350 (M^+^, 0.82), 75 (100). Anal. For: C_19_H_18_N_4_O_3_. Calcd. C, 65.14; H, 5.14; Nm 16.00; Found: C, 65.30; H, 5.25; N, 16.30.

*1-[3-Cyano-4-methyl-6-phenyl]-pyridine-2-yl-(2H)-phthalazine-3,8-dione* (**19**). A mixture of compound **5** (0.01 mol) and phthalic anhydride (0.01 mol) in acetic acid (20 mL) was refluxed for 6 h. The reaction mixture was concentrated, allowed to cool and poured onto H_2_O (100 mL). The solid obtained was recrystallized from dioxane. **19**: Yield 52%; m.p. 310–312 °C; IR cm^–1^: 3280 (NH), 3033 (CH–Ar), 2999 (CH–aliph.), 2222 (C≡N), 1739, 1714 (2C=O); ^1^H-NMR: δ 2.48 (s, 3H, CH_3_), 7.22–7.83 (m, 10H, Ar–H), 12.21 (brs,1H,NH); ^13^C-NMR: δ 11.82 (CH_3_), 186.98 (C=O), 202.65 (C=O), 118.76 (C≡N), 128.89–162.33 (Ar–C); MS *m/z* (%): 354 (M^+^, 1.08), 77 (100). Anal. For: C_21_H_14_N_4_O_2_: Calcd. C, 71.18; H, 3.95; Nm 15.81; Found: C, 71.30; H, 4.18, N, 15.50.

*8-Cyano-7-methyl-5-phenyl-2,3-dihydro-2-thioxo-1,2,4,triazolo-[4,3-a]-pyridine* (**20**). To a stirred suspension of compound 5 (22 mmol) in ethanol (20 mL), ethanolic potassium hydroxide (30 mL, 0.01 mol) and CS_2_ (2 mL) were added dropwise. The reaction mixture was then heated under reflux for 6 h. After cooling and evaporation of the solvent, the potassium salt obtained was dissolved in water and acidified with 2N aqueous HCl. The solid product formed was collected and recrystallized from ethanol. **20**: Yield 72%; m.p. 160–162 °C. IR cm^–1^: 3220 (NH), 3055 (CH–Ar) 2880 (CH–aliph.), 2220 (C≡N), 1644 (C=N); ^1^H-NMR: δ 2.48 (s, 3H, CH_3_), 6.88–7.45 (m, 6H, Ar–H) 11.33 (s, 1H, NH); ^13^C-NMR: δ 14.58 (CH_3_), 118.67 (C≡N), 212.00 (C=S), 125.78–148.79 (Ar–C); MS *m/z* (%) 266 (M^+^, 3.11) 75 (100). Anal. For: C_14_H_10_N_4_S. Calcd. C, 63.15; H, 3.75; N, 21.05; S, 12.03; Found: C, 63.30; H, 4.00; N, 21.30; S, 12.11.

## 4. Conclusions

New pyridine derivatives **4**, **5**, **11**–**13**, **18** and **19** were synthesized starting from compounds **4** and **5**. The present syntheses are one pot methods and afforded pyrido-[2,3-d]-pyrimidine and pyrazolo-[3,4-b]-pyridine derivatives in moderate to good yields. In addition, our study was extended to the synthesis of additional fused pyridine, such as isoquinolines **2a**,**b**, 1,8-naphthyridine **14** and 1,2,4-trizolo-[3,4-a]-pyridine derivative **20**. Spectral and analytical data of the newly synthesized compounds were all in good agreement with the proposed chemical structures.

## References

[1] Patel N.B., Agravat S.N., Shaikh F.M. (2011). Synthesis and antimicrobial activity of new pyridine derivatives-I. Med. Chem. Res..

[2] Patel N.B., Agravat S.N. (2009). Synthesis and antimicrobial studies of new pyridine derivatives. Chem. Heterocycl. Compd..

[3] Srivastava A., Pandeya S.N. (2011). “Indole” a versatile nucleuse in pharmaceutical field. Int. J. Curr. Pharm. Rev. Res..

[4] Paronikyan E.G., Noravyan A.S., Dzhagatspany I.A., Nazaryan I.M., Paronikyan R.G. (2002). Synthesis and anticovulsant activity of isothiazolo-[5,4-b]-pyrano (thiopyrano)-[4,3-d]-pyridine and isothiazolo [4,5-b]-2,7-naphthyridine derivatives. Pharm. Chem. J..

[5] Bernardino A.M.R., De Azevedo A.R., Pinheiro L.C.D., Borges J.C., Carvalho V.L., Miranda M.D., De Meneses M.D.F., Nascimento M., Ferreira D., Rebello M.A. (2007). Syntehsis and antiviral activity of new 4-(phenylamino)/4-[(methylpidin-2-yl) amino]-1-phenyl-1H-pyrazolo-[3,4-b]-pyridine-4-carboxylic acid derivatives. Med. Chem. Res..

[6] Tucker T.J., Sisko J.T., Tynebor R.M., Williams T.M., Felock P.J., Flynn J.A., Lai M., Liang Y., McGaughey G., Liu M. (2008). Discovery of 3-{5[(6-Amino-1*H*-pyrazolo-[3,4-b]-pyridine-3-yl)methoxy]-2-chlorphenoxy}-5-chlorobenzonitrile (MK-4965): A potent, orally bioavailable HIV-1 non-nucleoside reverse transcriptase inhibitor with improved potency against key mutant viruses. J. Med. Chem..

[7] Mamolo M.G., Zampieri D., Falagiani V., Vio L., Ferrone M., Pricl S., Banfi E., Scialino G. (2004). Antifungal and antimycobacterial activity of new *N*^1^-[1-aryl-2-(1*H*-imidazol-1-yl and 1*H*-1,2,4-triazol-1-yl)-ethylidene]-pyridine-2-carboxamidrazone derivatives: A combined experimental and computational approach. ARKIVOC.

[8] Gangjee A., Adair A., Queener O., Pneumo S.F. (1999). *Pneumocystis carinii* and *Toxoplasmo gondii* dihydrofoflate reductase inhibitors and antitumor agends: Synthesis and biological activities of 2,4-diamino-5-methyl-6-[(monosubstituted aniline)methyl]-pyrido [2,3-d] pyrimidines. J. Med. Chem..

[9] Geffken D., Soliman R., Soliman F.S.G., Abdel-Khalek M.M., Issa D.A.E. (2011). Synthesis of new series of pyrazolo[4,3-d]pyrimidin-7-ones and pyrido [2,3-d] pyrimidin-4-ones for their bacterial and cyclin-dependent kinases (CDKs) inhibitory activities. J. Med. Chem. Res..

[10] Nasr M.N., Gineinah M.M. (2002). Pyrido [2,3-d] pyrimidines and pyrimido [5′,4′:5,6] pyrido [2,3-d] pyrimidines as new antiviral agents: Synthesis and biological activity. Arch. Pharm..

[11] Golubev A.S., Starostin G.S., Ghunikhin K.S., Peregudov A.S., Rodygin K.C., Rubtsova S.A., Slepukhin P.A., Kuchin A.V., Chkamokov N.D. (2011). Synthesis of new fluorine-containing pyrazolo-[3,4-b]-pyridinones as promising drug precursors. Russ. Chem. Bull..

[12] De Clercq E. (2005). Recent highlights in the development of new antivirial drugs. Curr. Opin. Microbiol..

[13] Eizuru Y. (2003). Development of new antivirals for herpes viruses. Antivir. Chem. Chemother..

[14] Mellol H., Echevarria A., Bernardino A.M.R., Canto-Cavalheiro M., Leon L.L. (2004). Antileishmanial pyrazolopyridine derivatives: Synthesis and structure activity relationship analysis. J. Med. Chem..

[15] Tarik E.S.A. (2009). Synthesis of some novel pyrazolo-[3,4-b]-pyridine and pyrazolo [3,4-d] pyrimidine derivatives bearing 5,6-diphenyl-1,2,4-triazine moiety as potential antimicrobial agents. Eur. J. Med. Chem..

[16] Hassan A.Y., Ghorab M.M., Nassar O.M. (1997). Synthesis of certain new pyridine derivatives as potential radioprotective and cytotoxic agents. Indian J. Heterocycl. Chem..

[17] Smith T.P. (1995). Regioselective conversion of 3-cyano-6-hydroxy-2-pyridones into-3-cyano-6-amino-2-pyridones. J. Heterocycl. Chem..

[18] Stadlbauer W., Fiala W., Fisher M., Hojas G. (2000). Thermal cyclization of 4-azido-3-nitropyridines to furoxanes. J. Heterocycl. Chem..

